# Decidualization of endometriosis in a cohort of IVF-mediated pregnancies

**DOI:** 10.1038/s41598-022-05635-8

**Published:** 2022-01-27

**Authors:** Francesca Filippi, Laura Benaglia, Federica Alagna, Irene La Vecchia, Rossella Biancardi, Marco Reschini, Edgardo Somigliana, Paolo Vercellini

**Affiliations:** 1 Infertility Unit, Fondazione IRCCS Ca’ Granda Ospedale Maggiore Policlinico, Via M. Fanti, 6, 20122 Milan, Italy; 2 Centro Scienze Natalità, Gynecol/Obstet Unit, San Raffaele Scientific Institute, Milan, Italy; 3 Department of Clinical Sciences and Community Health, Università degli Studi di Milano, Milan, Italy; 4 Gynecology Unit, Fondazione IRCCS Ca’ Granda Ospedale Maggiore Policlinico, Milan, Italy

**Keywords:** Infertility, Urogenital reproductive disorders

## Abstract

Decidualization is the process of endometrial change in pregnancy, a phenomenon that can involve also ovarian endometriomas. However, the frequency of this event remains unknown. In addition, there is no evidence on the decidualization of deep invasive endometriosis (DIE). To shed more light on this issue, we prospectively recruited women with ovarian endometriomas or DIE who underwent IVF. They were subsequently excluded if they did not become pregnant or if they had a miscarriage. The evaluation was repeated in five time points during pregnancy and post-partum. The primary outcome was the rate of decidualized endometriomas at 11–13 weeks’ gestation. Data from 45 endometriomas and 15 nodules were available for data analyses. At the 11–13 weeks’ ultrasound, endometriomas’ decidualization was observed in seven cases, corresponding to 16% (95% CI 8–29%). Subsequent assessments in pregnancy failed to identify any additional case. DIE also underwent significant changes during pregnancy. At the 11–13 weeks’ ultrasound, lesions were increased in size and more vascularized. In conclusion, decidualization of ovarian endometriomas in IVF pregnancies is not rare. DIE may also undergo decidualization, but further evidence is needed for a robust and shared definition of this process.

## Introduction

The endometrium undergoes significant modifications in the secretory phase and in the beginning of pregnancy, a process generally referred as decidualization^1^. Stromal cells assume an epithelioid secretory aspect, the glandular epithelium becomes highly secretory, uterine natural killer (uNK) cells proliferate and macrophages propagate into the endometrium. These phenomena are governed by progesterone along with local paracrine factors and initiates soon after ovulation in stromal cells adjacent to blood vessels^1,2^. These transformations create conditions for implantation and development of early gestation.

In women with endometriosis, decidualization may also affect ectopic endometrium^3^. Decidualized ovarian endometriomas have been described as ovarian cysts with the typical ground glass or low level echogenity that, in addition, show vascularized papillary projections with smooth contours^4–7^. However, evidence from epidemiological studies aimed at assessing the accuracy of this definition is scant. The most informative contribution is a retrospective case series of 34 consecutive ovarian masses operated in pregnancy. The authors showed that all 12 women carrying lesions fulfilling these sonographic criteria were diagnosed with benign decidualized endometriomas. However, four additional cases of decidualized endometriomas did not show this pattern^6^. Overall, this evidence is insufficient for robust conclusions and the differential diagnosis between decidualized endometriomas and ovarian cancer (particularly borderline tumors) remains challenging^6,7^. This is clinically relevant because the management radically differs (expectant management with sonographic follow up for endometriomas, immediate surgery even during pregnancy for ovarian cancers). Improving our knowledge on decidualized endometriomas and more precisely estimating the frequency of this event may therefore be helpful for clinical practice. To date, available epidemiological information on this aspect is conflicting and biased. Only data from three retrospective studies focusing on adnexal masses diagnosed in pregnancy were published^8–10^. However, estimating the incidence of decidualization with this study design is exposed to confounders and may over-estimate the frequency. Prospective studies recruiting women before pregnancy are essential.

In addition, little is known about decidualization of deep infiltrating endometriotic (DIE) nodules^3,11^. We could not find a univocal description of the sonographic features of this process.

In the present study, we report on the sonographic follow up throughout pregnancy of women with endometriosis who achieved pregnancy with IVF. The primary aim of the study was estimating the frequency of sonographic decidualization of endometriomas. The secondary aim was providing a description of ultrasonographic modifications of DIE during pregnancy.

## Materials and methods

Women undergoing IVF cycles at the Infertility Unit of the Fondazione Ca’ Granda, Ospedale Maggiore Policlinico, Milan, Italy between January 2018 and December 2019 were prospectively evaluated for study entry. Inclusion criteria were the following: (1) age between 18 and 43 years, (2) indication to IVF, (3) presence of one or more endometriotic lesions (ovarian endometriomas or deep endometriotic nodules) at the basal transvaginal ultrasound performed prior to initiate the IVF cycle (lesions had to be documented in at least two US assessments at least 2 months apart), (4) acceptance to participate (written informed consent was obtained from all the participants). Women entering the study were subsequent excluded if they delayed the IVF cycle of more than 2 months, if they did not become pregnant, if they had a miscarriage before 12 weeks’ gestation or if they did not perform the assessment scheduled at 11–13 weeks’ gestation. Eligible women who were excluded could be re-considered if they entered another IVF cycle. Women could be included more than once if they had more than one pregnancy progressing beyond 12 weeks’ gestation. The study was approved by the local Institutional review board (Comitato Etico Milano Area 2). All methods were performed in accordance with the relevant guidelines and regulations.

At the time of enrolment (step 1), women underwent a transvaginal ultrasound to evaluate uterus position and morphology; myometrial features; presence, dimensions and characteristics of ovarian cysts; presence of sonographic soft markers of endometriosis (i.e. site-specific tenderness or fixed ovaries); presence of the “sliding sign” in the pouch of Douglas; presence, dimensions and characteristics of DIE nodules in the anterior and posterior compartments^12^. The evaluation of ovaries and DIE nodules was repeated in five more steps: at 6–7 weeks’ gestation (step 2), at 11–13 weeks’ gestation (step 3), at 23–25 weeks’ gestation (step 4), at 35–37 weeks’ gestation (step 5) and 30–40 days postpartum (step 6). The assessments at steps 4 and 5 were performed using both transvaginal and transabdominal probes. All the ultrasounds were performed by three expert sonographers (F.A., L.B. and F.F.) with International Ovarian Tumor Analysis (IOTA) certification. They were not blinded to the results of the previous assessments. If possible, women were exclusively scanned by the same sonographer in all the steps. Endometriomas were defined as round-shaped cystic masses with thick walls, regular margins and homogeneous low-echogenic fluid content with scattered internal echoes and mean diameter ≥ 10 mm^13^. Deep nodules were defined as hypoechoic lesions with irregular outer margins and few blood vessels within and around the nodules at Doppler examination^13^. No additional radiological investigations, including Magnetic Resonance Imaging, were done. Remarkable ultrasound images were saved so they could be reviewed and discussed with the other sonographers, if necessary. Mean diameter of the lesions was calculated as the mean of three perpendicular diameters. Vascularization was assessed by color Doppler imaging using a subjective semiquantitative assessment and refers to the whole lesion. A color score of 1 was given when no blood flow was found in the lesion; a color score of 2 when only minimal flow was detected; a color score of 3 when moderate flow was present, and color score of 4 when the lesion appeared highly vascular with marked blood flow. A papillary projection was defined as a solid projection into the cyst cavity from the cyst wall with a height ≥ 3 mm^14^. In all the assessments, women were requested to report any worsening of endometriosis-related symptoms (such as dyspareunia or pelvic pain).

In our Unit, infertile women with endometriosis were systematically counseled regarding the pros and cons of surgery and IVF, and a shared decision was taken^15^. Factors favoring IVF included previous surgery for the disease, abnormal semen, older age, bilateral endometriomas (because of the risk of severe postsurgical damage to ovarian reserve) and low ovarian reserve. Factors favoring surgery included pelvic pain (if refractory to progestins), large endometriomas (diameter > 4 cm), the presence of deep peritoneal lesions causing intestinal or urinary symptoms and non-reassuring imaging findings. Women with an indication to IVF were not scheduled for systematic surgery prior to initiate the cycle. During the IVF cycle, women were monitored and managed according to a standardized clinical protocol as reported in detail elsewhere^16^. Participation in the study did not modify the standard care in pregnancy.

Decidualization is reported to develop mostly in the first trimester^4^. Moreover, ovarian evaluation in the second and third trimester might not be always possible due to the presence of the pregnant uterus. For these reasons, the main outcome was the rate of decidualized endometriomas at 11–13 weeks’ gestation. Secondary outcomes were the evolution of decidualized endometriomas throughout pregnancy and postpartum and modifications of DIE nodules. If a patient had more than one endometrioma on the same ovary, we considered only the largest one. For DIE nodules, we exclusively considered those located in the posterior compartment since their visualization was simpler and deemed more reliable.

Definition of decidualization was previously established for endometriomas, the typical feature being the presence of rounded vascularized (color score ≥ 2) papillary projections^6^. Therefore, if a solid component with significant blood flow appeared in an endometriotic cyst at the 11–13 weeks’ ultrasound, we deemed the cyst as decidualized. We could not find any sonographic description of decidualized DIE nodules, hence we limited to describe changes in their volume and vascularization throughout pregnancy.

The sample size (about 40 endometriotic cysts) was calculated based on the following assumptions: (1) decidualization rate expected from previous studies: 12%^6,7^; (2) wideness of the 95% Confidence Interval (CI): 20% (± 10%).

Data was analyzed using the SPSS software 26.0 (Chicago, IL). Data is reported a mean ± Standard Deviation (SD), or median [interquartile Range (IQR)] or number (%), as appropriate. A binomial distribution model was used to determine the 95% CI of proportions. Student paired *t* test was used to compare lesion growth between the basal and the 11–13 weeks’ assessment. A mixed model was used to evaluate whether pregnancy impacted on the dimension of the lesions.

## Results

One hundred thirty-nine women were initially recruited, of whom 99 were subsequently excluded (87 did not become pregnant or delayed the IVF cycle of more than 2 months, nine had a first trimester miscarriage and three pregnant cases withdrew consent to participate). Forty pregnancies in 39 women who underwent at least basal and 11–13 weeks’ ultrasounds were included (one woman had two pregnancies). Baseline characteristics of the population are shown in Table 1. For fresh transfers, a GnRH antagonist protocol, a long protocol, and a flare-up protocol were used in 20, 6 and 2 cases, respectively. In all of them, supplementation was done with vaginal progesterone 90 mg daily for 2 weeks, up to the time of serum hCG assessment. For frozen embryo transfers, a pure natural cycle without adding any form of progesterone was used in 9 cases. The remaining 3 were treated with hormone replacement therapy. Thirty-eight pregnancies (95%) ended in a live birth, one in a pregnancy termination for a chromosomal anomaly, and one was lost to follow up after the 23–25 weeks’ ultrasonographic scan. None of the women required surgery in pregnancy because of endometriosis-related complications and none developed a spontaneous hemoperitoneum in pregnancy (SHiP). None reported worsening of their endometriosis-related symptoms during pregnancy.

**Table 1:** Baseline characteristics of the included women (n = 40).

Characteristics	Number (%), Median [IQR]
Age (years)	35 [31–37]
BMI (kg/m^2^)	20.8 [19.6–23.5]
Previous deliveries	5 (13%)
AMH (ng/mL)	2.3 [1.3–4.4]
AFC	12 [4–17]
Previous surgery for endometriosis	12 (30%)
Hormonal treatment prior to IVF	12 (30%)
**Type of embryo transfer**	
Fresh	28 (70%)
Frozen	12 (30%)

One woman was included twice.

*IQR* interquartile range, *BMI* Body Mass Index, *AMH* anti-mullerian hormone, *AFC* antral follicle count.

Overall, data from 45 endometriotic cysts and 15 nodules were available for analyses. Eight women had bilateral endometriotic cysts and three had DIE without endometriomas. Basal characteristics of the endometriotic lesions are summarized in Table 2.

**Table 2:** Baseline characteristics of the endometriotic lesions.

Characteristics	Number (%), Median [IQR]
**Endometriomas (n = 45)**	
Mean diameter (mm)	23 [17–31]
Endometriomas larger than 20 mm	29 (64%)
Papillations	6 (13%)
Papillations with color score ≥ 2	0 (0%)
**Deep peritoneal nodules (n = 15)**	
Mean diameter (mm)	14 [13–29]
Color score ≥ 2	0 (0%)

These characteristics refer to the baseline situation, i.e. before initiating the ovarian hyper-stimulation.

*IQR* interquartile range.

**Figure 1:**
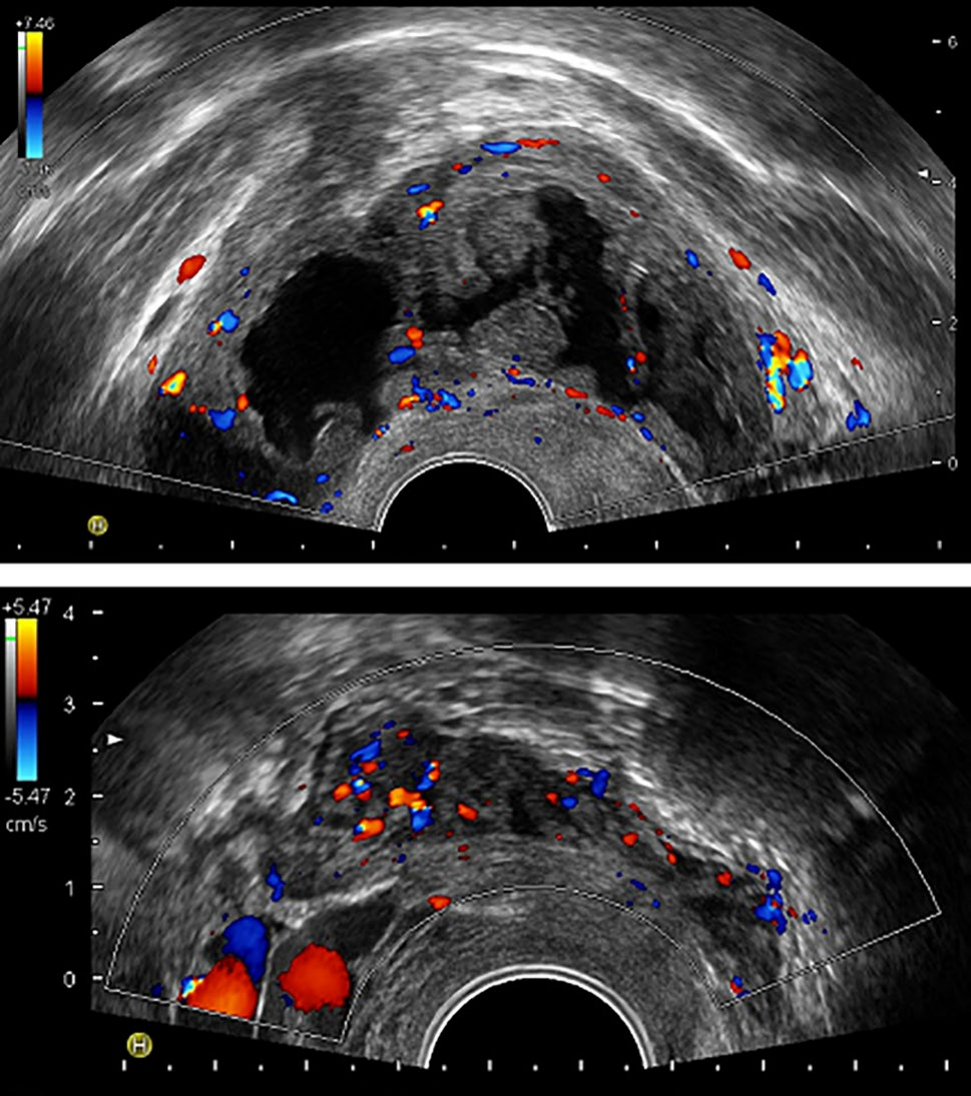
Decidualization of ovarian endometriomas and deep peritoneal nodules. In the upper panel, a decidualized endometrioma at 12 weeks’ gestation is shown (Case 6 left, see Table 3). Multiple vascularized papillary vegetations can be observed. Their color score was quoted as 2. In the lower panel, a retrocervical endometriotic nodule at 23 weeks’ gestation is represented (Case 15, see Table 4). The mean diameter of the lesion increased from basal evaluation to 23 weeks’ gestation from 25 to 29 mm. Doppler evaluation showed a color score of 3 (at baseline it was 1).

When comparing the mean diameter of the endometrioma between basal and 11–13 weeks’ assessments, no significant difference emerged (25 ± 10 vs 24 ± 15 mm, respectively, p = 0.63). Seven endometriomas in six women developed vascularized papillary projections at the 11–13 weeks’ ultrasound and were deemed decidualized (16%, 95% CI 8–29%). Figure 1 illustrates one of these cases. Signs of decidualization could not be observed at 6–7 weeks’ gestation assessment in any of these seven cases. Three decidualized endometriomas had multiple (three to four) papillary projections and a significant growth of the cyst (increase in diameter of more than 50%). The remaining four had a single vascularized papillary projection and a grossly stable cystic dimension. Among the eight women with bilateral endometriomas, six had no decidualization, one had unilateral decidualization and one had bilateral decidualization. The woman included twice carried endometriomas in both pregnancies and never showed signs of decidualization. Decidualization exclusively occurred in women who had fresh embryo transfer (6/28) whereas it was never observed among those who had frozen embryo transfer (0/12). However, the difference was not statistically significant (Fisher Exact test, p = 0.15). The six women showing decidualization were treated with a GnRH antagonist protocol in 4 cases, a long protocol in 1 case, and a flare-up protocol in 1 case.

Thirty-six women (90%) underwent the 23–25 weeks’ ultrasound, 32 women (80%) the 35–37 weeks’ ultrasound and 33 women (82%) the post-partum ultrasound. We failed to document new cases of decidualization after the 11–13 weeks’ assessment. The presence and modification of sonographic signs of endometriomas’ decidualization during the study period is presented in detail in Table 3. Vascularized papillary projections regressed after delivery in all cases. None of the seven included cysts had a significant volume growth after 11–13 weeks’ gestation. Supplemental Table 1 presents data for non-decidualized endometriomas. When considering post-partum assessments in all women with endometriomas (data available for 29 women, corresponding to 36 cysts), lesions were no more detectable in 13 cases (36%). When comparing the rate of endometriomas’ disappearance between cysts which did and did not undergo decidualization, a statistically significant difference was found: 0/7 (0%) compared to 13/29 (45%), p = 0.03. When exclusively consider endometriomas that could be detected both before and after pregnancy (n = 25), a significant reduction in size emerged. The median [IQR] diameter dropped from 23 [16–31] to 17 [13–21] mm (p = 0.001). The mixed model analysis on all the assessments aimed at capturing a general impact of pregnancy on the dimension of the endometrioma resulted statistically significant (p < 0.001).

**Table 3:** Decidualization of endometriomas during pregnancy.

Cases	Step 1		Step 2		Step 3		Step 4		Step 5		Step 6	
	Pre-pregnancy		6–7 weeks		11–13 weeks		23–25 weeks		35–37 weeks		Post-partum	
	VP	Mean diameter	VP	Mean diameter	VP	Mean diameter	VP	Mean diameter	VP	Mean diameter	VP	Mean diameter
Case 1	−	27	−	32	+	30	−	27	−	21	−	19
Case 2	−	42	−	31	+	31	−	38	−	16	−	20
Case 3	−	20	−	20	+	25					−	12
Case 4	−	16	−	16	+	12	−	13	−	26	−	8
Case 5 left	−	32	−	37	+	50	+	44		*Hidden*	−	22
Case 6 right	−	18	−	19	+	42	+	44		*Hidden*	−	14
Case 6 left	−	22	−	35	+	58	+	47		*Hidden*	−	24
Median diameter	22 [18–32]		31 [19–35]		31 [25–50]		41 [24–45]		21 [16–26]		19 [12–22]	

Only the seven cases who showed typical signs of decidualization in at least on of the assessments are included in the table.

*VP* vascularized papillary projection. Present (+) or absent (−).

Hidden means that the lesion could not be seen because of the pregnancy. Empty boxes mean that the woman did the not perform the assessment.

Mean diameter of the endometrioma is reported (in mm). Median diameter is presented with the interquartile range.

Four of the 15 DIE nodules could not be identified at the 11–13 weeks’ ultrasound as well as in subsequent scans in pregnancy. One of these four cases occurred in the patient who had bilateral decidualized endometriomas. However, in this case, we cannot exclude that we were just unable to distinguish the DIE nodule because it was in close contact with the two enlarged kissing ovaries carrying the decidualized endometriomas. To note, in this case, the DIE nodule could be easily identified at post-partum assessment, when decidualization of the endometriomas regressed completely. In contrast, in the other three cases that could not be identified in pregnancy, the DIE nodule could not be detected in the post-partum. The analyses were therefore made for the 11 nodules that could be identified at both the basal and the 11–13 weeks’ ultrasound scans. Findings are detailed in Table 4. A significant increase is size emerged: the mean diameter grew from 16 ± 4 to 20 ± 6 mm (p = 0.001). At the 11–13 weeks’ ultrasound, five nodules (45%) showed an increase in blood flow (color score changed from 1 to 2 in four nodules and from 1 to 3 in one nodule). One of these cases is illustrated in Fig. 1. As opposed to what described for endometriomas, we observed changes also after the 11–13 weeks’ assessment. An increase in color score later in pregnancy was documented in three additional nodules (27%). DIE lesions could not be identified in two out of the nine women (22%) who performed the post-partum evaluation. When exclusively consider DIE lesions that were detected both before and after pregnancy (n = 7), no modification in size emerged, the median [IQR] diameter before and after pregnancy being 15 [13–19] and 18 [12–24] mm, respectively (p = 0.35). The mixed model analysis aimed at capturing a general impact of pregnancy on the dimension of the DIE lesion did not detect a statistically significant effect (p = 0.10).

**Table 4:** Modifications of deep invasive endometriosis during pregnancy.

Cases	Step 1		Step 2		Step 3		Step 4		Step 5		Step 6	
	Pre-pregnancy		6–7 weeks		11–13 weeks		23–25 weeks		35–37 weeks		Post-partum	
	CS	Mean diameter	CS	Mean diameter	CS	Mean diameter	CS	Mean diameter	CS	Mean diameter	CS	Mean diameter
Case 3	1	20	1	26	1	23					1	14
Case 7	1	13	1	17	1	17	1	34	3	30		
Case 8	1	13	1	12	1	13	*Not seen*		*Not seen*		*Not seen*	
Case 9	1	17	1	23	1	22	2	22	1	33	1	20
Case 5	1	12	1	13	1	16	1	13	1	21	*Not seen*	
Case 11	1	17	1	18	2	18	1	22	3	24	2	25
Case 12	1	14	1	13	2	13	1	22	3	18	1	14
Case 13	1	14	2	20	1	20	1	19	2	30	1	8
Case 14	1	13	1	23	1	18	1	17	1	20	1	17
Case 15	1	25			3	32	3	29	3	32	1	24
Case 16	1	20	1	22	2	28	2	36	2	29		
Median diameter	14 [13–20]		19 [13–23]		18 [16–23]		22 [18–32]		29 [21–31]		17 [14–24]	

Only cases whose DIE lesions could be identified at least in the 12 weeks’ assessment are included in the table.

Cases are not reported in numerical order because we first presented women who also had a decidualized endometrioma and we wanted to respect the numbering given in Supplemental Table 1. From case 7 to case 13, women did not have decidualized endometriomas. From case 14 onwards, women did not carry endometriomas at all.

*CS* color score.

Mean diameter of the lesions is reported in mm. Median diameter is presented with the interquartile range.

## Discussion

Decidualization of ovarian endometriomas in pregnancy is not rare. In our prospective study, this phenomenon occurred in seven out of 45 endometriomas, corresponding to 16% (95% CI 8–29%). In addition, our study confirms previous findings suggesting that decidualization of ovarian cysts essentially develops during the first trimester of pregnancy, remains steady or regress during the second trimester of pregnancy and consistently disappears after delivery^3,4,9^.

Three previous retrospective studies reported on the incidence of endometriomas’ decidualization. Our results are in line with the rates reported by Ueda et al*.*^8^ and Pateman et al.^9^, but higher that what reported by Bailleux et al.^10^. Combining all these three reports with our findings (in total 15 decidualizations out of 151 endometriomas) allows to estimate that the frequency of decidualization would be about 10% (95% CI 6–16%), i.e., one out of 10 cases.

The awareness that endometrioma decidualization occurs in 16% of cases should be kept in mind when vascularized projections suddenly develop at the beginning of pregnancy in women knowing to carry ovarian endometriomas. Decidualization rather than cancer degeneration is the most plausible explanation in these cases and women should be reassured. Indeed, all seven cases described in our series were benign. Similarly, in the case series of Mascillini et al., all 12 ground-glass ovarian cysts with vascularized smooth papillations were found to be decidualized endometriomas^6^. However, this element may be less valid in natural compared to IVF pregnancies because women who conceive spontaneously are not always aware of carrying an endometrioma. In these cases, an in-depth sonographic evaluation in referral centers is needed to minimize misdiagnoses^6^.

The natural history of DIE nodules in pregnancy partly differed from ovarian endometriomas. Firstly, in four cases, we were unable to identify the lesions. The short time interval between the basal and first assessment that was done at 6–7 weeks’ gestation tends to rule out the possibility of resorption of the lesions. We initially interpreted this evidence as due to a modification of the echogenicity, a change that can impair the capacity to distinguish them from the surrounding organs. However, this explanation contrasts with the observation that lesions could not be detected in three cases after delivery. A false positive diagnosis at basal ultrasound is an alternative explanation that we cannot exclude^17^. Secondly, we observed a significant growth of the lesions and an enhanced vascularization in a one third (5 out of 15) of DIE cases. Even if our statistical power is insufficient for robust conclusions, the frequency of these changes appears more frequent than for endometriomas. Thirdly, these modifications tend to progress over pregnancy rather than self-limiting at the end of the first trimester, as observed for endometriomas. This is in line with the epidemiology of spontaneous hemoperitoneum in pregnancy, a rare but frightful complication of pregnancy that is mainly considered as a complication of DIE decidualization^15,18–20^. This condition indeed tends to occur later in pregnancy, generally in the second part of gestation. Interestingly, Coccia et al. studied three women with DIE during pregnancy and reported a progressive growth of the nodules up to 24 weeks’ gestation, but then a progressive regression^9^. No data was reported on vascularization. A precise definition of sonographic DIE decidualization is lacking in the literature and cannot be drawn from our study. A valid definition would need to be based on clinical consequences, i.e., the definitions of sonographic changes that are associated with increased risk of pregnancy complications^19,20^.

The occasional disappearance of lesions at post-partum evaluation is an additional and interesting finding of our study. Endometriomas and DIE lesions could not be detected in 36% and 22% of cases, respectively. These results are in line with previous evidence that, however, exclusively referred to endometriomas^4,21^. We also observed that this disappearance was more common among cysts that did not undergo decidualization. To note, we cannot exclude that our inability of detecting lesions could also be influenced by the proximity of delivery (the assessment was made 30–40 days after delivery). A definite recover to the non-pregnant condition may not have completely occurred at this time. A long-term follow-up 6–12 months after delivery would be necessary. More in general, the issue of the possible lesions’ disappearance with pregnancy warrants specific investigations. If confirmed, this element should also be considered in the decision-making regarding the role of surgery prior to IVF. Finally, it is worth noting that, for lesions that could be assessed both before and after pregnancy, we observed a reduction in size for endometriomas but not for deep lesions. This is intriguing but the small sample size hampers robust conclusions. Further investigations are needed.

Decidualization of endometriosis in pregnancy is enigmatic and intriguing. Why do most changes occur in the first trimester? Why does decidualization occur in some but not all lesions? Why has this phenomenon never been described in non-pregnant women who are treated with continuous progestins for long period of time? Our study cannot address these important queries. Nonetheless, the observation that in a woman with bilateral endometriomas decidualization was observed in only one cyst suggests that this event is lesion- rather than patient-specific. Similarly, we observed women who had DIE modifications (increase in size or vascularization) but not endometrioma decidualization, and the other way round. One may speculate that this could depend on the relative component of endometrial cells and fibrosis within the lesions^22^. Possibly, decidualization could occur also in cases with higher fibrotic component but being so limited to be undetectable at ultrasound. Another possibility is that endometriotic cells of different lesions may differently respond to pregnancy hormones. One may speculate that older lesions could be less sensitive. Alternatively, one may consider the possible role of progesterone resistance, a common phenomenon in endometriosis that could explain the “resistance” of most lesions to the increase in pregnancy-related progesterone. More in general, one could theoretically expect decidualization to occur more frequently, if not always. But this would not take place because of progesterone resistance. This phenomenon is known for a long time but is recently receiving renewed attention^23–26^. This pathogenetic mechanism could also explain the variable and unpredictable impact on lesions’ dimension observed in our cohort over the whole pregnancy.

Other important clinical queries require future investigation. Of particular interest is the identification of predictive factors of decidualization. Identifying pre-pregnancy clinical or sonographic characteristics that expose women to an increased risk of decidualization would be clinically relevant. This information could help deciding between expectant management or prophylactic surgery before pregnancy. In this regard, it is worth noting that there is a growing consensus on opting directly to IVF for women with endometriosis without preliminary treating the disease^27–29^. This clinical attitude is gaining credit because of the observation that endometriotic lesions are generally unremarkable to IVF success and rarely progress with ovarian hyperstimulation^30,31^. Unfortunately, our sample size was insufficient to draw any meaningful information on predictive factors. To note, in our series, all the decidualized endometriomas were observed among women who had fresh embryo transfer (thus being exposed to ovarian hyperstimulation) and none among those achieving pregnancy with frozen embryos. Even if this difference was not statistically significant, this observation merits utmost attention. If confirmed, this could have practical implications. One could consider transferring only frozen embryos. To note, there is some biological evidence supporting this possibility^32,33^. Jarvela et al. showed that serum progesterone is markedly higher in pregnancies obtained with fresh embryo transfer during the whole first trimester. Serum estrogens are also increased even if this elevation ends at about 8 weeks’ gestation^32^. One may speculate that this perturbed endocrine milieu may favour decidualization of endometriosis. Another argument suggesting a potential detrimental effect of ovarian hyperstimulation is the observation that spontaneous hemoperitoneum in pregnancy (thought to be related to endometriosis’ decidualization) is more frequent in IVF pregnancies^15,19,20^.

Some limitations of our study deserve to be commented. First, we lack the histological confirmation of the diagnosis of decidualization, as none of the included patients underwent surgery for endometriosis during the follow-up. We also lack histological confirmation of endometriosis. However, this second limitation is of doubtful relevance given the elevated accuracy of transvaginal ultrasound. The sensitivity and specificity of this diagnostic tool for ovarian endometriomas are 93% (95% CI 87–99%) and 96% (95% CI 92–99%), respectively^34^. For DIE in the rectovaginal septum, sensitivity and specificity are 0.59 (95% CI 0.26–0.86) and 0.97 (95% CI 0.94–0.98), respectively^35^. For DIE in the uterosacral ligaments, they are 0.67 (95% CI 0.55–0.77) and 0.86 (95% CI 0.73–0.93), respectively^35^. Second, reliability of ultrasounds to monitor ovarian lesions during pregnancy is not validated. This limitation is particularly relevant for the assessments performed in the second part of pregnancy because of the presence of the enlarged uterus that can hide the lesions or complicate their visualization. In fact, the transabdominal approach was commonly required in advanced pregnancy. Third, all the included pregnancies were achieved with IVF. This gave us the possibility to have an ultrasound evaluation just before pregnancy, thus providing accurate evaluations. However, caution is warranted prior to generalize our conclusions to the whole population of women with endometriotic lesions. We cannot exclude a synergistic effect of ovarian hyper-stimulation (and thus the presence of multiple corpora lutea) and pregnancy.

In conclusion, decidualization of ovarian endometriomas in pregnancy is not rare. A correct diagnosis is essential to avoid useless and possibly harmful surgery. In addition, our study shows that also DIE nodules can undergo important modifications during pregnancy that can be detected at ultrasounds, in particular the increase in vascularization. Further evidence is needed to clarify the possible additive effect of ovarian hyper-stimulation and to draw a shared definition of DIE decidualization.

## Supplementary Information

Supplementary Table 1.
