# Supplementary material for: Decidualization of endometriosis in a cohort of IVF-mediated pregnancies
Source: Sci Rep. 2022 Jan 27;12:1524. doi: 10.1038/s41598-022-05635-8 (PMC8795262; doi:10.1038/s41598-022-05635-8)
Supplement: Supplementary file 1 — Supplementary Table 1. [file 41598_2022_5635_MOESM1_ESM.doc]

| **Supplemental Table 1.** Modifications of endometriomas during pregnancy. | | | | | | | | | | |  |  |  |  |  |  |  |
| --- | --- | --- | --- | --- | --- | --- | --- | --- | --- | --- | --- | --- | --- | --- | --- | --- | --- |
|  |  |  |  |  |  |  |  |  |  |  |  |  |  |  |  |  |  |
| Cases | Step 1 | |  | Step 2 | |  | Step 3 | |  | Step 4 | |  | Step 5 | |  | Step 6 | |
|  | Pre-pregnancy | |  | 6-7 weeks | |  | 11-13 weeks | |  | 23-25 weeks | |  | 35-37 weeks | |  | Post-partum | |
|  | VP | Mean diameter |  | VP | Mean diameter |  | VP | Mean diameter |  | VP | Mean diameter |  | VP | Mean diameter |  | VP | Mean diameter |
|  |  |  |  |  |  |  |  |  |  |  |  |  |  |  |  |  |  |
| Case 5 right | - | 21 |  | *not seen* | |  | *not seen* | |  | *not seen* | |  | *not seen* | |  | *not seen* | |
| Case 7 | - | 32 |  | - | 30 |  | - | 28 |  | *not seen* | |  | *not seen* | |  |  | |
| Case 8 | - | 17 |  | - | 19 |  | - | 15 |  | - | 12 |  | *not seen* | |  | *not seen* | |
| Case 9 right | - | 15 |  | - | 18 |  | - | 17 |  | - | 16 |  | - | 22 |  | - | 21 |
| Case 9 left | - | 24 |  | - | 24 |  | - | 22 |  | *not seen* | |  | *not seen* | |  | *not seen* | |
| Case 10 | - | 11 |  | - | 11 |  | - | 9 |  | - | 14 |  | - | 26 |  | *not seen* | |
| Case 11 | - | 23 |  | - | 21 |  | - | 31 |  | - | 31 |  | - | 32 |  | - | 12 |
| Case 12 right | - | 21 |  | - | 38 |  | - | 40 |  | - | 18 |  | *not seen* | |  |  | |
| Case 12 left | - | 23 |  | - | 24 |  | - | 23 |  | - | 20 |  | *not seen* | |  |  | |
| Case 13 | - | 24 |  | - | 19 |  | - | 17 |  | - | 19 |  | *not seen* | |  | *not seen* | |
| Case 17 right | - | 15 |  | - | 17 |  | - | 14 |  | *not seen* | |  | *not seen* | |  | *not seen* | |
| Case 17 left | - | 17 |  | - | 13 |  | - | 15 |  | - | 12 |  | *not seen* | |  | - | 29 |
| Case 18 | - | 33 |  | - | 32 |  | - | 30 |  | - | 36 |  |  | |  |  | |
| Case 19 | - | 22 |  | - | 23 |  | - | 22 |  | - | 23 |  |  | |  | - | 14 |
| Case 20 right | - | 17 |  | - | 15 |  | - | 15 |  | *not seen* | |  | *not seen* | |  | *not seen* | |
| Case 20 left | - | 11 |  | - | 12 |  | - | 11 |  | - | 22 |  | *not seen* | |  | - | 21 |
| Case 21 | - | 20 |  | - | 25 |  | - | 22 |  | - | 39 |  | - | 22 |  | - | 20 |
| Case 22 | - | 25 |  | - | 27 |  | - | 12 |  | *not seen* | |  | - | 21 |  | - | 11 |
| Case 23 | - | 44 |  | - | 35 |  | - | 33 |  | - | 25 |  | - | 21 |  | - | 21 |
| Case 24 | - | 37 |  | - | 32 |  | - | 29 |  | - | 26 |  | - | 34 |  | *not seen* | |
| Case 25 right | - | 13 |  | - | 13 |  | - | 25 |  | *not seen* | |  | *not seen* | |  | - | 14 |
| Case 25 left | - | 31 |  | - | 34 |  | - | 12 |  | - | 12 |  | *not seen* | |  | *not seen* | |
| Case 26 right | - | 23 |  | - | 22 |  | - | 10 |  | *not seen* | |  |  | |  | - | 8 |
| Case 26 left | - | 16 |  | *not seen* | |  | - | 10 |  | *not seen* | |  |  | |  | *not seen* | |
| Case 27 | - | 31 |  | - | 30 |  | - | 30 |  | - | 13 |  | - | 34 |  | - |  |
| Case 28 | - | 58 |  | - | 73 |  | - | 74 |  | - | 86 |  | - | 68 |  | - | 39 |
| Case 29 | - | 11 |  | - | 13 |  | - | 13 |  | - | 6 |  | - | 10 |  | *not seen* | |
| Case 30 | - | 14 |  | *not seen* | |  | - | 12 |  | *not seen* | |  |  | |  | *not seen* | |
| Case 31 | - | 16 |  | - | 13 |  | - | 10 |  |  | |  |  | |  |  | |
| Case 32 | - | 16 |  | - | 18 |  | - | 10 |  |  | |  | *not seen* | |  | *not seen* | |
| Case 33 | - | 29 |  | - | 25 |  | - | 20 |  |  | |  | - | 16 |  | - | 17 |
| Case 34 | - | 44 |  | - | 51 |  | - | 51 |  | - | 22 |  | - | 19 |  | - | 18 |
| Case 35 | - | 22 |  | - | 14 |  | - | 20 |  |  | |  |  | |  |  | |
| Case 36 | - | 41 |  | - | 40 |  | - | 42 |  | - | 8 |  | *not seen* | |  | - | 17 |
| Case 37 | - | 25 |  | - | 19 |  | - | 12 |  | - | 11 |  |  | |  |  | |
| Case 38 | - | 25 |  | - | 25 |  | - | 9 |  | - | 16 |  | - | 12 |  | - | 14 |
| Case 39 | - | 32 |  |  | |  | - | 31 |  | - | 19 |  | *not seen* | |  |  | |
| Case 40 | - | 28 |  | - | 30 |  | - | 31 |  | - | 27 |  | - | 30 |  | - | 33 |
|  |  |  |  |  |  |  |  |  |  |  |  |  |  |  |  |  |  |
| Median diameter | 23 [16 - 31] | |  | 24 [17 - 31] | |  | 20 [12 - 30] | |  | 19 [12 - 26] | |  | 22 [18 - 33] | |  | 18 [14 - 21] | |
|  |  |  |  |  |  |  |  |  |  |  |  |  |  |  |  |  |  |
|  |  |  |  |  |  |  |  |  |  |  |  |  |  |  |  |  |  |
| Only the cases who not showed typical signs of decidualization are included in the table. | | | | | | | | | | | | | | | | | |
| VP: Vascularized Papillary projection. Present (+) or absent (-). | | | | | | | | | | | | | | | | | |
| Empty boxes mean that the woman did the not perform the assessment. | | | | | | | | | | | | | | | | | |
| Mean diameter of the endometrioma is reported (in mm). | | | | | | | | | | | | | | | | |  |
